# Genomic signatures of local adaptation to the degree of environmental predictability in rotifers

**DOI:** 10.1038/s41598-018-34188-y

**Published:** 2018-10-30

**Authors:** Lluis Franch-Gras, Christoph Hahn, Eduardo M. García-Roger, María José Carmona, Manuel Serra, Africa Gómez

**Affiliations:** 1 Institut Cavanilles de Biodiversitat i Biologia Evolutiva, Universitat de València, A.O.22085, 46071 Valencia, Spain; 2 School of Environmental Sciences, University of Hull, HU6 7RX Hull, United Kingdom

## Abstract

Environmental fluctuations are ubiquitous and thus essential for the study of adaptation. Despite this, genome evolution in response to environmental fluctuations —and more specifically to the degree of environmental predictability– is still unknown. Saline lakes in the Mediterranean region are remarkably diverse in their ecological conditions, which can lead to divergent local adaptation patterns in the inhabiting aquatic organisms. The facultatively sexual rotifer *Brachionus plicatilis* shows diverging local adaptation in its life-history traits in relation to estimated environmental predictability in its habitats. Here, we used an integrative approach —combining environmental, phenotypic and genomic data for the same populations– to understand the genomic basis of this diverging adaptation. Firstly, a novel draft genome for *B. plicatilis* was assembled. Then, genome-wide polymorphisms were studied using genotyping by sequencing on 270 clones from nine populations in eastern Spain. As a result, 4,543 high-quality SNPs were identified and genotyped. More than 90 SNPs were found to be putatively under selection with signatures of diversifying and balancing selection. Over 140 SNPs were correlated with environmental or phenotypic variables revealing signatures of local adaptation, including environmental predictability. Putative functions were associated to most of these SNPs, since they were located within annotated genes. Our results reveal associations between genomic variation and the degree of environmental predictability, providing genomic evidence of adaptation to local conditions in natural rotifer populations.

## Introduction

Environmental fluctuations —either predictable or unpredictable– occur to a varying extent in any locality, so that adaptation to the degree and type of environmental fluctuation is a critical need for an organism to persist in a locality. Interestingly, climate change, over-exploitation, and habitat alteration often cause not just an environmental novelty in the average conditions, but an increase of fluctuations, with an important unpredictable component^1–3^. Different evolutionary responses are expected to result from adaptation to environmental fluctuations and their degree of predictability^4^. One of these, phenotypic plasticity, occurs when the individuals modify their phenotype accordingly to environmental conditions, without genetic change^5^. Another well-studied response is adaptive tracking, in which environmental variation results in correlated variation in mean population traits, since natural selection may favour different phenotypes over evolutionary time by acting on the heritable variation in the population^6–8^. When organisms have dormant stages such as seed banks or diapausing egg banks^9,10^ adaptive tracking may result in the maintenance of high levels of genetic and ecologically relevant diversity in populations^11^, which could provide strong signatures of balancing selection in genomes. A third response, bet hedging, occurs when a single genotype produces different phenotypes (i.e. diversified bet hedging, “don’t put all the eggs in the same basket”) or a “low-risk” phenotype (i.e. conservative bet hedging, “a bird in the hand is worth two in the bush”), so that it lowers the variance in its reproductive success but maximizes the reproductive success in the long-term^12^. It has been suggested that the evolution of bet hedging and phenotypic plasticity could involve regulatory genes in the genome^8,13,14^. However, to date, few molecular mechanisms underlying some of these evolutionary strategies have been identified, and these were mainly in relation to phenotypic plasticity (e.g. in *Daphnia sp*.^15,16^). With the advent of cost-effective genomic technologies, it is now possible to screen hundreds or thousands of genes and assess which ones are diverging in their allele frequencies, in order to assess how they affect evolutionary fitness in the wild^17^. The ultimate objective is to map multilocus genotypes to phenotypes to environments^18,19^. Therefore, from a fundamental and applied perspective, it is important to know how genomes respond to environmental fluctuations —and more specifically to unpredictable environments^20–22^–, a topic still scarcely investigated.

The Mediterranean region typically exhibits very different environmental regimes in habitats even within relatively small geographical scales^23^. In particular, Mediterranean saline lakes are strongly seasonal and temporally unpredictable environments at several time scales^24^. In addition, these habitats are also affected by global processes, such as climate change or the North Atlantic Oscillation^25^. Given the lack of detailed long-term, *in situ* records of most of these habitats, Franch-Gras *et al*.^26^ analysed long-term trends in water-surface area using a 27 years long series of remote sensing data on water-surface area to characterize the relative importance of environmental fluctuations in a set of saline lakes in eastern Spain. The indices available to estimate the degree of predictability from time-series data, basically, decompose the time series based on periodic and stochastic variation^27^ and associate these with predictable and unpredictable fluctuations, respectively. Franch-Gras *et al*.^26^ estimated the water-surface area of each lake in each satellite image and calculated the predictable and unpredictable components of variation of that data by using different models that reflected the effect of fluctuations on different types of organisms. Thus, predictability is defined as the organism’s ability to anticipate and adjust to future environmental conditions^26^. Franch-Gras *et al*.^28^ concluded that the predictability metrics based on Colwell’s approach^29,30^ and on the presence/absence of water were the most appropriate for small aquatic invertebrates^28^ (see methods for more details). Remarkably, the saline lakes studied showed a wide range of predictability, when predictability was assessed taking into account the point of view of small aquatic invertebrates^26^.

Cyclically parthenogenetic rotifers are common small invertebrates in ponds and lakes. These passively-dispersed aquatic organisms have been used successfully to study the evolutionary responses to environmental fluctuations described above^31–33^. These rotifers combine clonal proliferation with occasional bouts of sexual reproduction which results in the production of diapausing eggs. These eggs are long-lived, and are able to persist through adverse environmental conditions, including drought. In this temperate region, rotifer populations are temporary —not being active all year round– and colonize the water column during the so-called growing season. Thus, sex and the concomitant diapausing-egg production are critical for population persistence. In the case of organisms established as clones hatching after a sexual reproduction phase (i.e. cyclical parthenogenesis), such as aphids, rotifers and cladocerans, genetic slippage due to a phase of clonal selection followed by sexual reproduction can result in phenotypic traits changing in a direction even opposite to selection^34^. In addition, rotifer diapausing egg banks maintain high levels of standing genetic variance for life history traits^35–37^, which indicates that they should have a high potential to adapt to environmental fluctuations using adaptive tracking^8^. Moreover, theory predicts low-risk strategies to occur in rotifer populations inhabiting unpredictable habitats that would consist on (1) early production of diapausing eggs^38^ and/or (2) intermediate hatching fraction of diapausing eggs^39^. Indeed, rotifer populations show signatures of local adaptation to salinity and temperature in their life history traits^35–37^. Recently, Franch-Gras *et al*.^28^ found high within-population genetic variation in two life-history traits associated to diapause in nine populations of the rotifer *Brachionus plicatilis* inhabiting saline lakes with varying degrees of environmental predictability^26^. These traits were: (1) the propensity for sexual reproduction^35,40^ as a proxy of the timing of diapausing egg production^41^; and (2) the diapausing egg hatching fraction (the lower, the longer the diapause duration). The propensity for sex differed among populations and was inversely correlated with lake predictability, suggesting a conservative bet-hedging strategy that would protect against unexpectedly short growing seasons.

The study of the genetic basis of adaptation in non-model organisms has recently been fuelled by the rapid development of genomic technologies, especially next generation sequencing^42–44^. One of these technologies, genotyping by sequencing (GBS)^45^ is a cost-effective technique to screen genome-wide patterns of diversity by using restriction enzymes to reduce the genome complexity and has been used successfully to identify genomic regions under selection in several non-model animals^46–48^. It allows the genotyping of a high number of individuals and the discovery of typically large numbers of single nucleotide polymorphisms (SNPs).

Here, we take advantage of such a well-characterized study system of populations to study the genomic basis of local adaptation to environmental features. We studied the genome-wide patterns of genetic variability and differentiation in response to environmental predictability using GBS in the same set of *B. plicatilis* clones studied in Franch-Gras *et al*.^28^ in populations characterised for their environmental predictability by Franch-Gras^26^. Since no *B. plicatilis* genome was available, whole genome sequencing data were obtained in order to assemble the first draft genome for this species with a structural and functional annotation. This genome was used as a reference to facilitate (1) GBS analysis of population structure and (2) discovery of putative loci under selection. We investigated the correlation of loci under selection to life-history traits and environmental variables and identified genes associated to these loci. In this study we firstly, gather evidence of genomic association to local adaptation patterns by considering several factors; and then we focus on the degree of environmental predictability as a factor having a wide range in the studied lakes^26^ and to which the inhabiting populations have been shown to present patterns of local adaptation^28^.

## Results

### Draft genome

A total of 11.7 Gb of raw genomic sequence data were obtained. Based on k-mer statistics (k-mer length = 21; Supplementary Fig. S1), *B. plicatilis* HYR-1 genome size was estimated to be 115.77 Mb with an heterozygosity of 0.65%. Out of the four assemblers used, the Platanus assembly showed the best features, with the highest N50, no evidence of contamination and high proportion of complete and partial gene sequences in CEGMA completeness report (Table 1). Therefore downstream analyses were performed using this assembly as reference. Genome completeness analysis by CEGMA found 88.7 and 96.4% of complete and partial sequences respectively of the set of core eukaryotic genes included in the analysis (Table 1). The assembly included 108.5 Mb of genomic sequence scaffolds with 50% of all bases in scaffolds longer than 20.4 Kb (N50) and a maximum scaffold length of 169.9 Kb (Table 2). Finally, Blobtools did not find evidence of contamination based on both G + C content and contig coverage (Table 1; Supplementary Fig. S2).

**Table 1:** Assembly features resulting from the different genome assemblers assayed (see text for details).

Assembler	Contamination (Blobtools)	N50 (in Kb)	Longest Scaffold (in Kb)	Number of Scaffolds	CEGMA % complete genes	CEGMA % partial genes	Reference
Celera	−	18.7	99.2	10,626	62.9	66.53	Myers *et al*. (2000)
DISCOVAR	+	14.0	219.0	29,076	89.1	97.18	Weisenfeld *et al*. (2014)
Platanus	−	20.4	170.0	14,326	88.7	96.37	Kajitani *et al*.^51^
SPAdes	+	15.4	731.9	31,020	91.94	95.56	Bankevich *et al*. (2012)

**Table 2:** Summary for *Brachionus plicatilis* draft genome assembly and gene annotation.

Number of scaffolds	14326
Total length (Mb)	108.5
Longest scaffold	169985
Scaffold %GC	26.4
Number of predicted genes	54725
Genes with functional annotation	16674

The structural gene annotation yielded a predicted set of 54,725 gene models, some 30% of which could be functionally annotated.

### GBS raw data, SNP calling and filtering

A total of 52 Gb of raw sequence data were obtained for the 270 clones from the nine populations. Out of the 630,460,573 raw reads, 379,536,357 quality-filtered barcoded reads were obtained. The SNP calling pipeline yielded 12,856 SNPs, resulting in 4,543 SNPs after filtering. Hardy-Weinberg proportions for each SNP and population showed that most SNPs do not differ from Hardy-Weinberg expectations (99.9% SNP with *p*-value > 0.05 after Bonferroni correction).

### Genome-wide population differentiation

**Figure 1:**
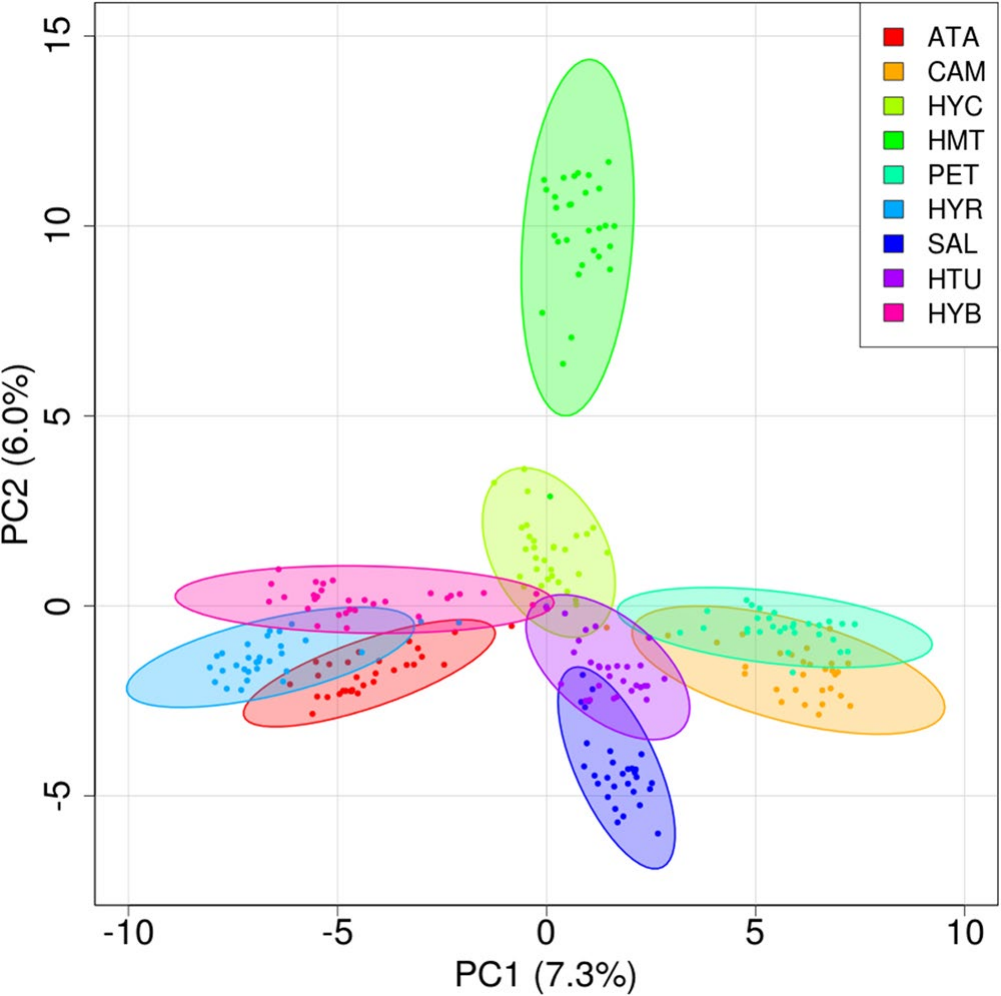
Principal component analysis based on 4,543 genome-wide SNPs for 30 clones (dots) from each of the nine *Brachionus plicatilis* populations. Ellipsoids are the 95% confidence interval for each population. Percentage of variance explained by each principal component is shown between parentheses in the axis label.

The overall expected heterozygosity (averaged over loci) was 0.17 and the within population expected heterozygosity ranged from 0.15 to 0.23 (Table 3). Heterozygosity correlated significantly to the log-transformed lake size (*R*^2^ = 0.40; *t* = 2.15, *df* = 7, *p*-value = 0.034). The overall *F*_*ST*_ (averaged over loci) was 0.18. Pairwise *F*_ST_ averaged across loci (Table 4) ranged from 0.07 (HYB and HYC) to 0.18 (SAL or HTU and HMT). The two major axes in PCA explained a joined variance of 13.1%. In the ordination space of these axes (Fig. 1), clones of the same populations were clustered, with some overlap between populations. There was not a significant pattern of isolation by distance (*r* = 0.31, *p*-value = 0.07, Mantel’s test).

**Table 3:** Population values of heterozygosity (*Ho*), expected heterozygosity (*He*) and inbreeding index (*F*_*IS*_) averaged over SNPs.

Population	*Ho*	*He*	*F*_*IS*_
PET	0.23	0.23	−0.014
SAL	0.17	0.17	0.009
ATA	0.17	0.17	0.025
HYR	0.15	0.15	0.000
HYC	0.20	0.19	−0.017
CAM	0.17	0.18	0.045
HMT	0.15	0.15	−0.016
HYB	0.17	0.17	0.009
HTU	0.15	0.15	0.012

**Table 4:** Population pairwise fixation index (*F*_*ST*_) values.

	ATA	CAM	HYC	HMT	PET	HYR	SAL	HTU	HYB
ATA									
CAM	0.13								
HYC	0.09	0.08							
HMT	0.16	0.15	0.10						
PET	0.13	0.07	0.08	0.13					
HYR	0.11	0.15	0.10	0.17	0.13				
SAL	0.13	0.12	0.10	0.18	0.12	0.15			
HTU	0.14	0.12	0.11	0.18	0.13	0.15	0.15		
HYB	0.09	0.14	0.07	0.14	0.13	0.08	0.13	0.12

### Putative SNPs under selection

**Figure 2:**
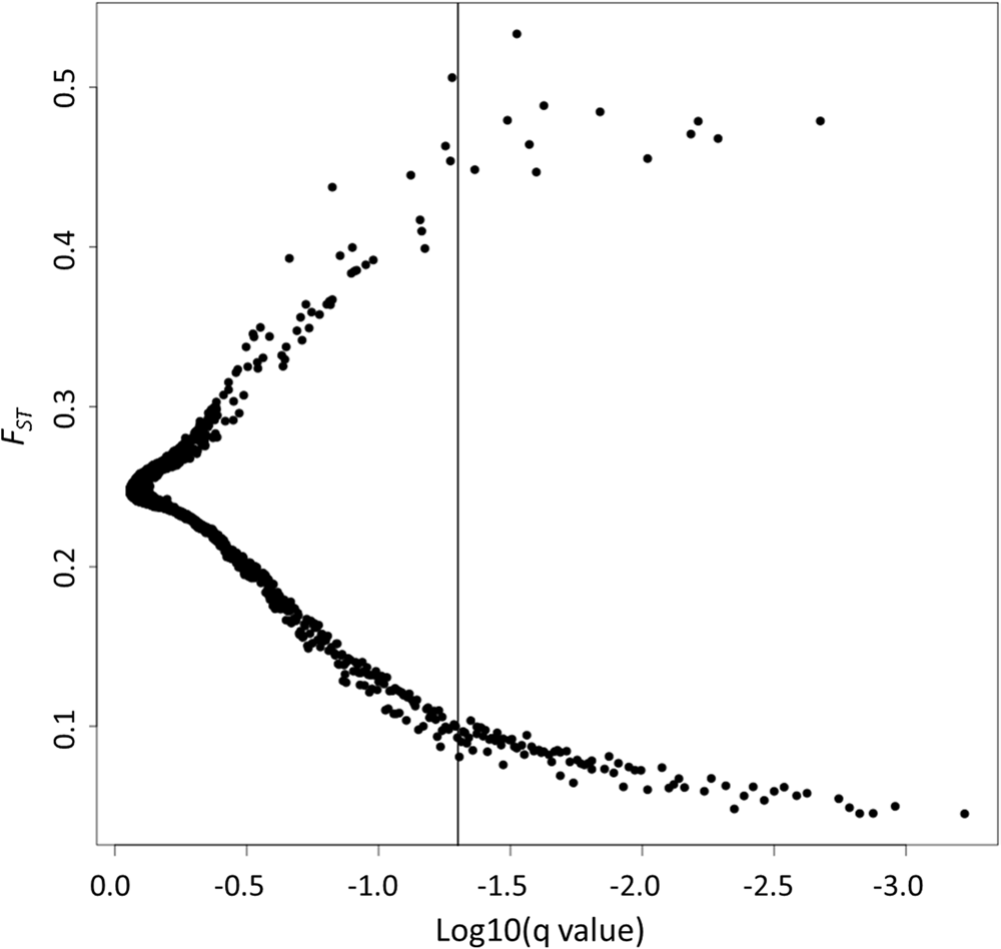
Relationship between *F*_*ST*_ and log10(*q* value) based on 4,543 genome-wide SNPs in nine rotifer field populations according to BayeScan. Adopting a *p*-value < 0.05 criterion, SNPs on the right side of the vertical line are putatively under selection.

Using the BayeScan approach, 93 SNPs within 66 genomic regions (i.e. scaffolds) were identified as candidates to be under selection. 12 of these SNPs had significantly higher *F*_*ST*_ than the background of the genome (i.e. they are putatively under diversifying selection) and 81 have significantly lower *F*_*ST*_ than the background of the genome (i.e. they are putatively under balancing/purifying selection; Fig. 2). The latter group of SNPs were found to have a similar coverage than the rest of the SNPs in the genome (Supplementary Fig. S3), ruling out an artifact of merging duplicated loci. In addition, we identified further signatures of balancing rather than purifying selection as of these 81 SNPs, 64 (79.0%) have higher minor allele frequencies than the mean of the rest of the SNPs in the genome (i.e. MAF > 0.145) and 68 (83.9%) have positive Tajima D values.

**Figure 3:**
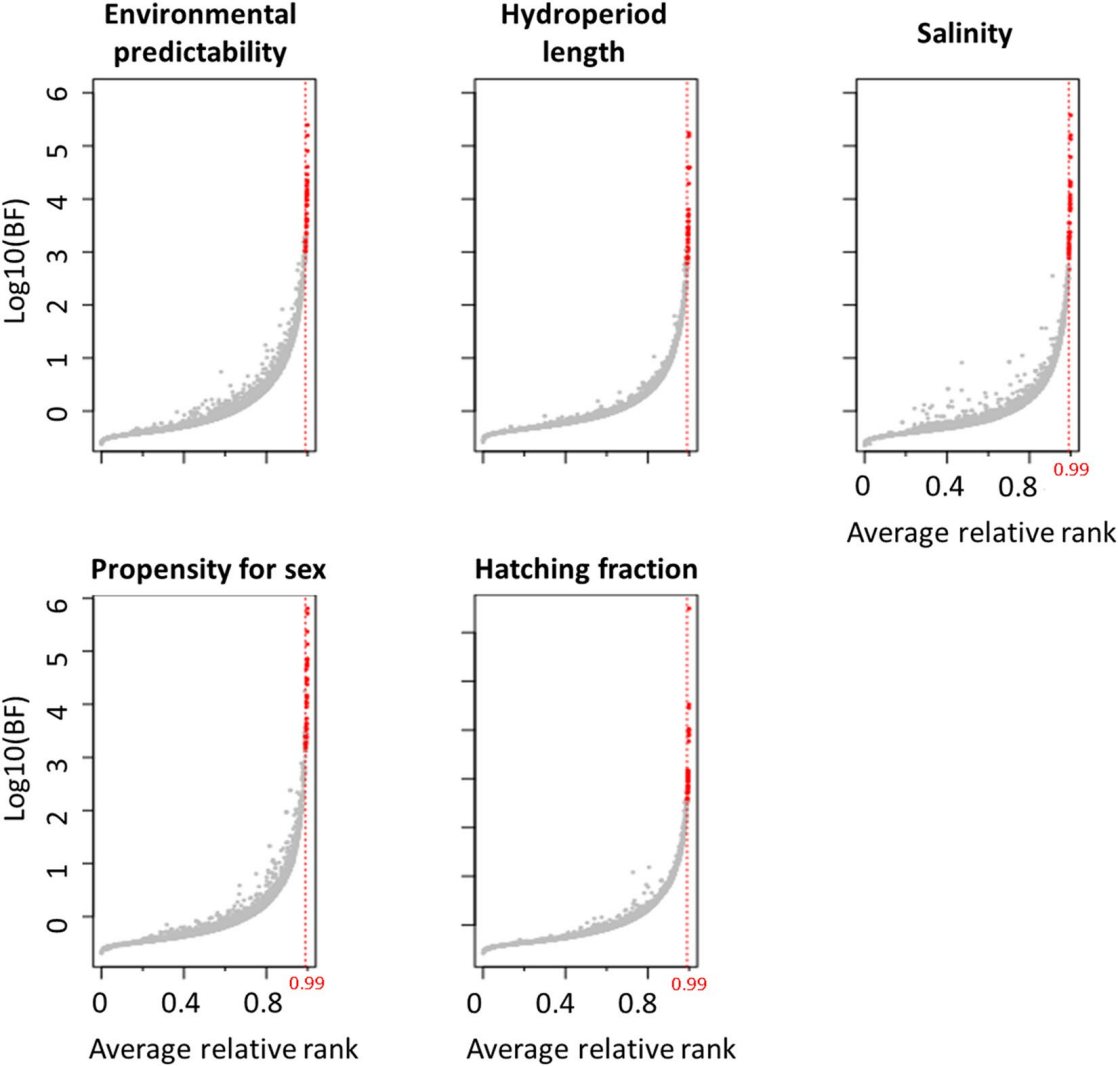
Bayesian factors (BF) and mean rank for the correlation between SNPs (dots) and three environmental (upper row panels) and two phenotypic (lower row panels) variables. Results are based on twenty replicate runs of Bayenv. Vertical red line shows the mean rank threshold (mean rank > 0.99) to consider a SNP to be outlier (red dots).

**Figure 4:**
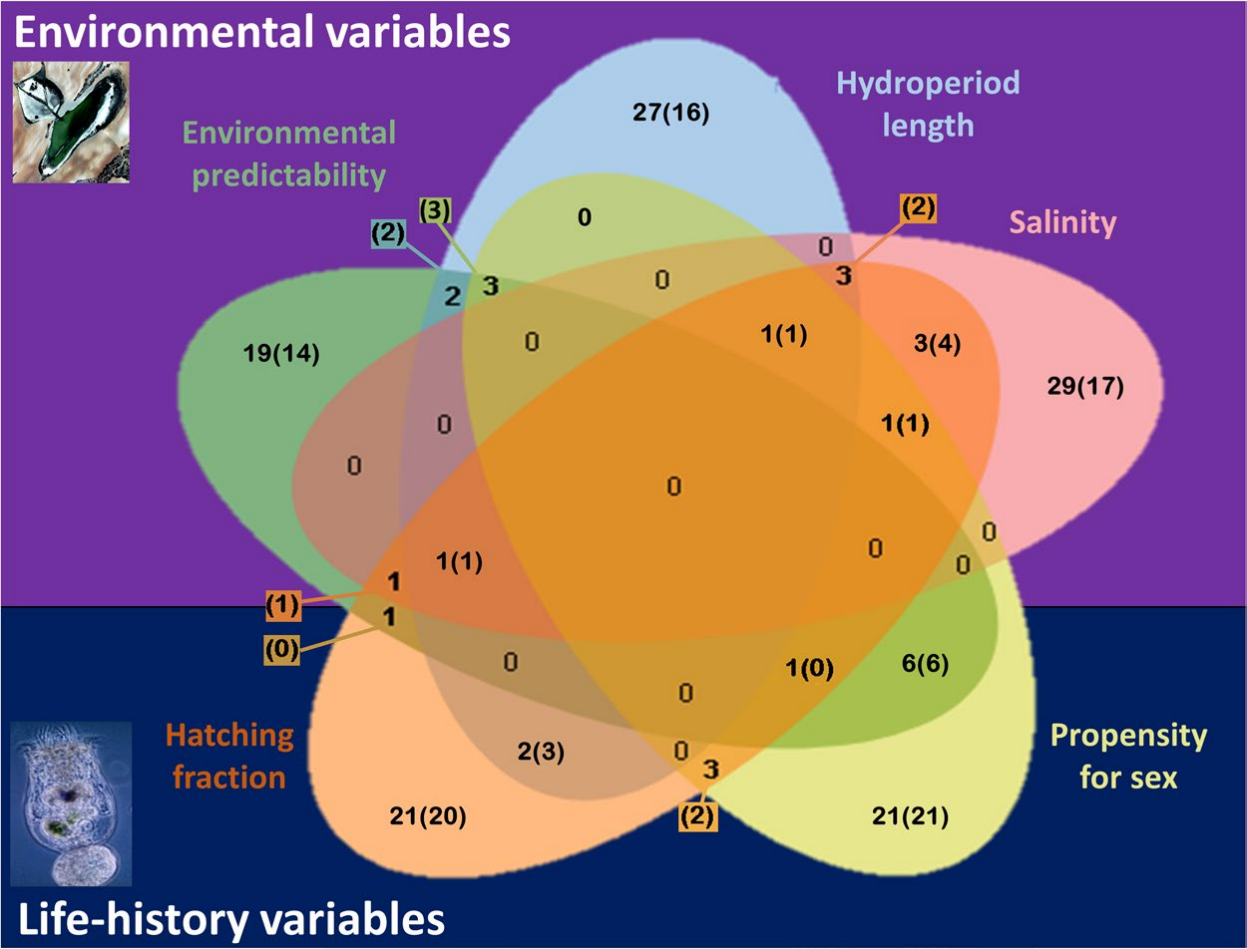
Venn diagram showing the number of SNPs correlated with each variable according to Bayenv. The number of genes with at least a SNP in its coding regions is shown between parentheses. Aerial image of the pond from PNOA 2009 CC-BY 4.0 Instituto Geográfico Nacional - ign.es.

Using the Bayenv approach, significant correlation with environmental and phenotypic variables was found for a number of SNPs ranging from 34 to 39 (Fig. 3). The highest Bayes factors were found for salinity, propensity for sex and hatching fraction. The highest number of SNPs correlated with two variables in our analysis (Fig. 4) were 10 SNPs located in ten genes (between hatching fraction and salinity) and 10 SNPs located in 9 genes (between propensity for sex and the degree of environmental predictability). Three (33.33%) of the SNPs detected by BayeScan as being under diversifying selection were also found strongly correlated with one of the variables tested in Bayenv (two to hatching fraction, one to propensity for sex and one to salinity; one of them was correlated with both salinity and hatching fraction see Supplementary Table S1). None of the SNPs putatively under balancing/purifying selection was also found to be correlated to any variable tested in Bayenv.

The GWAS analysis using GenABEL yielded a single SNP significantly associated to the propensity for sex, this SNP was not detected by Bayenv nor by BayeScan. No SNP was significantly associated to hatching fraction.

### Genes associated to SNPs putatively under selection

Most of the SNPs detected with BayeScan or Bayenv were located within genes (Supplementary Table S2). Out of the 12 SNPs that BayeScan detected under diversifying selection, 11 (91.7%) were located within 12 genes; the one that was not found within any gene was located at 1,783 bp from the closest gene. Regarding Bayenv, the number of genes ranged from 27 to 35. Finally, the only SNP detected by GenABEL (associated to propensity for sex) was within the coding region of a gene annotated as “Exosome complex exonuclease RRP44”.

The 164 putative genes under selection by either BayeScan, Bayenv or GenABEL (Supplementary Table S2) had a broad range of gene ontology (GO) annotations. According to the enrichment analysis, the genes associated to propensity for sex and environmental predictability were enriched for genes involved in binding, those associated to salinity were enriched in genes implicated in regulation of cellular processes; and those genes associated to hatching fraction were enriched in signal transducer activity (see details in Supplementary Table S3) For those genes associated to hydroperiod length, no significant gene ontology enrichment was found. The gene found to be related to propensity for sex in GenABEL was found to be associated to the following functions: rRNA catabolic process (GO:0006401), regulation of RNA metabolic process (GO:0051252), rRNA processing (GO:0006364), and mRNA catabolic process (GO:0006402).

## Discussion

We used a powerful, integrated approach —combining environmental, phenotypic and genomic data for the same set of populations– to identify genomic regions putatively under selection to local conditions, focusing on environmental predictability in rotifer populations. Moreover, we report a new draft genome assembly for *B. plicatilis*, in a taxonomic group (monogonont rotifers) in which the available genetic resources are still scarce (but see *Brachionus calyciflorus* genome)^49^. We identified candidate regions for diversifying selection, some of them are correlated with environmental predictability and some of these are correlated with life-history traits and other ecological factors. Moreover, we found a large set of genes showing signatures of balancing selection in rotifer populations.

### Draft genome of *Brachionus plicatilis*

We have described and annotated the first draft genome assembly of *B. plicatilis*. The estimated (haploid) genome size is 115.77 Mb, similar to the assembly size obtained (108.5 Mb) and the previous estimates for different *B. plicatilis* strains using flow-cytometry (between 111.7–128.8 Mb)^50^. The k-mer analysis revealed that the heterozygosity of the *B. plicatilis* genome is relatively high (0.65%). This is consistent with the finding that Platanus assembler yielded better results than other assemblers, since it is known to have a good performance when assembling highly heterozygous genomes^51^. The genome was highly compact, with a large number of predicted gene models (54,725). This compactness produced a high number of genes physically associated to the SNPs putatively under selection. Therefore the analysis was restricted to those genes where these SNPs were in their coding regions. Although this approach might potentially produce a loss of information, since introns and inter-genic space changes can also affect gene expression, the fast linkage decay with physical distance found after a linkage disequilibrium analysis (Supplementary Fig. S4) supports the use of such a restricted area of influence of the SNPs detected in our study species. Although *Brachionus plicatilis* is regarded as a model species for evolutionary biology studies^52,53^, before this contribution the available genetic resources for *B. plicatilis* were limited to EST libraries (NCBI database) and transcriptomes of congeneric species (Supplementary Table S4). Furthermore, there are only two rotifer genomes available: (1) the distantly related bdelloid *Adineta vaga*, belonging to the strictly asexual class Bdelloidea^54^ and (2) the recently published *Brachionus calyciflorus* genome^49^. It would be desirable both to improve the quality of the genome assembly, for instance, by the inclusion of both more sequencing data (such as mate-pairs or PacBio platform sequencing) and gene mapping information and transcriptome data.

### Genes and SNPs putatively under selection

We identified 12 candidate genes under diversifying selection in the nine rotifer populations studied using Bayescan. These results indicate that rotifer genomes show signatures of adaptation to local conditions (not necessarily to predictability). Previous work has shown local adaptation in life history traits at intermediate (29,000 km^2^; Campillo *et al*.)^37^ and small (240 km^2^; Franch-Gras *et al*.)^28^ spatial scales, in the Mediterranean region. *Brachionus plicatilis* populations in Spain harbour a high degree of ecologically relevant genetic variation frequently involving sexual reproduction and diapause strategies^35,55,56^. This variation has been found to be correlated with environmental features such as salinity^55^ and environmental predictability^28^. However, the genetic basis of local adaptation in this populations remained unknown. There are very few studies considering the genetic basis of local adaptation in aquatic invertebrates. Orsini *et al*.^57^ related several genes of *Daphnia magna* to a set of natural anthropogenic stressors using an experimental evolution approach, and identified repeatable patterns of local adaptation. Roulin *et al*.^16^ identified one gene in the same species associated to a photoreceptor inducing a plastic response of diapause that is involved in local adaptation patterns.

We are aware that *F*_*ST*_ methods such as Bayescan have low power when populations are high differentiated for neutral markers^58,59^, which is the case for *B. plicatilis* (see below), however we used a powerful correlation analysis (Bayenv) to identify candidate SNPs strongly associated with environmental predictability, hydroperiod length, salinity and two life history traits related to diapause. This correlation-based analysis uses information of important environmental and phenotypic variables for each lake/population, which are not considered in the *F*_*ST*_ method (BayeScan), resulting in the ability to detect both adaptive responses to each variable or genes that might determine diapausing-related traits. Three out of the 12 SNPs found to be putatively under diversifying selection by BayeScan were also highlighted as strongly correlated by Bayenv, what highlights the importance of the studied variables in driving local rotifer adaptation. Therefore these three SNPs are very strong candidates to be under diversifying selection in response to hatching fraction, propensity for sex and salinity. In addition, the GWAS analysis using GenABEL yielded one SNP significantly associated to the propensity for sex.

From those SNPs that Bayenv related to the variables, 10 (located in ninegenes) were correlated simultaneously to both the propensity for sex and the degree of environmental predictability (Fig. 4). As a result, 29.4% of the SNPs highlighted by Bayenv as correlated with the degree of predictability were also correlated with the propensity for sex. This provides a genetic basis to the tight relationship between these two variables, according to the findings in Franch-Gras *et al*.^28^. In addition, the weak relationship between the degree of predictability and the hatching fraction observed by Franch-Gras *et al*.^28^ is consistent with our results at the genomic level. Only four SNPs detected by Bayenv (located in two genes) were correlated with both variables simultaneously. This implies that only 11.7% of the SNPs correlated with the degree of predictability were also correlated with the hatching fraction. The 37 SNPs found to be correlated with propensity for sex in Bayenv plus the one obtained by GWAS and the 38 SNPs found to be correlated with the hatching fraction are strong candidates to be underlying the genomic basis of each of these traits.

Though our main interest was on unravelling the genomic responses to unpredictable environments, we also explored the effects of lake area and geographic distance on population genetic parameters, and adaptive responses to other environmental parameters such as hydroperiod length and salinity, as they are likely additional factors to explain adaptive genetic divergence among populations or can act as confounding factors. Our results agree with the findings by Franch-Gras^28^ regarding the low influence of hydroperiod length on the propensity for sex once the effect of environmental predictability is discounted.

A large number of SNPs that are correlated with the studied environmental variables (i.e. hydroperiod length, environmental predictability or salinity) do not correlate with life-history traits (i.e. propensity for sex or hatching fraction), suggesting that the former acted as selective pressures on traits not considered here. Conversely, a high number of SNPs correlated with the life-history traits studied were not correlated with the environmental variables. Obviously, not all the genetic variability associated to these fitness components is responding to selection by these particular environmental variables. Other selective pressures not considered here —such as lake trophic status or temperature– could be acting. Nonetheless, through adaptive tracking, genes causing divergence in life-history traits might be responding to short-term fluctuations that are uncoupled across localities. Genetic differentiation in *Daphnia* populations caused by this selective scenario has been reported^11^. Interestingly, fluctuations of water-surface area —an important feature and a proxy for other features such as variations in the physicochemical conditions are poorly correlated among the lakes studied^26^. Most of the SNPs correlated with any of the variables studied here are located within genes, which showed a range of gene ontologies, so that reliable inferences are difficult. However, a specific insight on gene functionality was achieved for the genes associated to the propensity for sex and environmental predictability, because the set showed an enrichment of genes that have been putatively associated to binding functions. Interestingly, both phenotypic plasticity and bet hedging have been proposed to be regulated by epigenetic mechanisms^13,14^. In addition, genes associated to hatching were found to be enriched in signal transducer activity and genes associated to salinity to regulation of cell cycle or of biological processes. After examining individual gene functions, some interesting putative functions of genes associated to specific variables are discussed below. Indeed, a gene associated to hatching fraction is described as “coronin-7 isoform X1” and is involved in the actin filament polymerization, which is upregulated in diapausing mosquitos^60^. A gene associated to hydroperiod, described as “estrogen receptor beta”, which increases reproduction when being activated by a ligand in the rotifer *Brachionus manjavacas*^61^. Moreover, a gene associated to predictability is described as “POU class transcription factor 1-like”, a transcription factor which is more expressed in non-diapause than in diapause pupae in the moth *Helicoverpa armigera*^62^. A gene associated to propensity for sex in this study described as “speedy A” has a “male meiotic nuclear division” GO term, as is involved in reproductive meiosis in mice^63^ and pigs^64^. Finally, a gene associated to salinity is described as “G2 mitotic-specific cyclin partial” and has a “regulation of cell cycle” GO term, the arrest of the cell cycle in diapause has been associated to the G2 stage in *Bombyx mori*^65^. Although tantalizing, these are tentative gene function descriptions and caution should be taken when considering these results. Undoubtedly, additional information on rotifer genome and transcriptomics would refine the functionality of genes that are candidates to be targets of selection by the degree of environmental predictability.

We identified a high proportion of putative SNPs under selection with signatures of balancing or purifying selection, 54 out of the 66 genomic regions (i.e. scaffolds) under selection. We found that most of these SNPs (74.1%) had both minor allele frequencies higher than the genome average and positive Tajima D values, suggesting that they are under balancing rather than purifying selection or being false positives^66^. In addition, we confirmed that this result is not due to artifactually over-merging duplicated genes (Supplementary Fig. S3). Although finding evidence of genomic regions under balancing selection is not unexpected^67–69^, it is unusual to find such a large number of genomic regions under balancing selection within a small geographical area. Several mechanisms have been proposed to underlie balancing selection, including heterozygote advantage^70^, negative frequency-dependent selection^71^, temporal habitat heterogeneity^72,73^, genetic slippage^34^ or conflicting optimal fitness between life-cycle stages^74^. The fact that the clones used in this study were established from diapausing egg banks may also be relevant, as such egg banks can accumulate diversity relevant to different selection pressures in different time periods^9,75^ despite not showing differences in neutral markers^75^. Although further research is needed to determine the mechanisms underlying the observed pattern of balancing selection, temporal habitat heterogeneity (including fluctuating selection patterns) or loci with conflicting fitness effects in different life-cycle stages^35^ could be important in the studied *B. plicatilis* populations. Indeed, these results are consistent with the high polymorphism of *B. plicatilis* genomes despite prolonged clonal phases of proliferation of their populations^35^, with the large intrapopulation genetic diversity for ecologically relevant life history traits^28,33,37^. The GWAS analysis allowed this inter-clonal variability to be analysed, and yielded one SNP associated to propensity for sex.

Given that we used a genome representation technique (Genotyping by sequencing, GBS), which does not screen all available SNPs in the genome, our analysis resulted in an average distance between SNPs is 25.73 kb (115.77 Mb/4500), which is over the distance at which the linkage disequilibrium drops (see Supplementary Fig. S4). Thereby, it is likely that this study does not present a comprehensive list of loci involved in local adaptation. Further explorations of the SNPs of the genome of *B. plicatilis* are needed in order to find out further SNPs involved in adaptation to local conditions, especially when the whole genome sequencing techniques become affordable enough to apply them to a large set of individuals in population genetics studies.

### Population structure

Genome-wide patterns of genetic variation did not reveal a geographically based population structure, which was not unexpected given the proximity of the studied populations. In previous studies, isolation-by-distance has been reported in this species at a larger regional scale^55,76^, but —consistently with what was found in the same geographical area using microsatellites and two mitochondrial genes by Montero-Pau *et al*.^77^– no evidence was observed here. The overall population differentiation (*F*_*ST*_ = 0.18) was slightly lower than the estimate obtained by Montero-Pau *et al*.^77^ with microsatellite markers (*F*_*ST*_ = 0.25) for the same geographical area. Consistently to what Montero-Pau *et al*.^77^ observed using mitochondrial data, we found a significant relation between genetic diversity at the genomic scale (measured as estimated heterozygosity) and lake size, suggesting that lake size is a reliable proxy for effective population size in *B. plicatilis* populations.

## Conclusions

We have used a powerful approach in a set of natural rotifer populations integrating genomic data with data on environmental features of their habitats^26^ and their phenotypic diversity^26^. Our genomic analysis identifies and screens a large number of high quality SNPs (4,543) in a small —but dense in gene regions– genome, assembled and annotated here. Some of these SNPs were putatively under selection, with strong signatures of both diversifying and balancing selection. Moreover, a large number of SNPs were also correlated with environmental predictability and other environmental and phenotypic variables. Remarkably, most of these SNPs were located within functionally annotated genes on the genome. Our results provide evidence of genomic adaptive responses to local conditions in natural rotifer populations, focusing on the degree of environmental predictability.

## Material and Methods

### Samples and study sites

**Figure 5:**
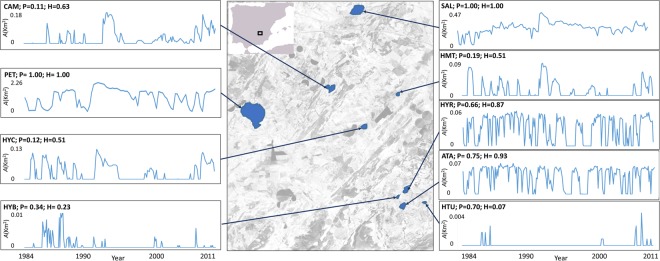
In the center, location of the nine lakes studied here. For each pond the water-surface area time series and its predictability (*P*) and hydroperiod values (*H*) are shown. Data obtained from Franch-Gras *et al*.^26^.

*Brachionus plicatilis* clones from nine Spanish salt lakes differing in predictability conditions (Fig. 1, Table 5) were obtained from diapausing egg banks in a previous study^28^. A total of 30 clones from each field population were established and maintained under standard laboratory conditions (Supplementary Method S1). Data on the degree of environmental predictability, hydroperiod length and size of the lakes are from Franch-Gras *et al*.^26^, data on the life-history traits from the *B. plicatilis* clones are from Franch-Gras *et al*.^28^. Briefly, the predictability metric was calculated in Franch-Gras *et al*.^26^ by constructing a contingency table (with two rows: presence/absence of water; and 12 columns: the calendar months) with the complete time series of each lake. With this contingency matrix, as described in Colwell^29^ and Stearns^30^, predictability was calculated as the summation of two values: constancy and contingency. The time-series for water-surface area and the resulting predictability values associated to each pond are shown in Fig. 5. Salinity is an important parameter shaping rotifer life-history traits^55,78^ and the Mediterranean region salt lakes have a wide range of salinity^24,79^, therefore salinity was also measured during the sampling of the lake sediments (May-September 2013). Note that this salinity measure is based on a single visit, thus although it is a value that may be indicative of the salinity of each of the ponds it does not integrate its temporal variability.

**Table 5:** Variables of the studied lakes and populations (obtained from Franch-Gras *et al*.^26,28^).

Lake/population	Acronym	Lake variables				Population variables	
		Area (m^2^)	Salinity (g/L)	Hydroperiod length	Environmental predictability	Propensity for sex (ind./mL)	Hatching fraction (%)
Pétrola	PET	1190000	18.68	1.00	1.00	8.3	44.2
Salobralejo	SAL	237000	6.3	1.00	1.00	8.3	76.8
Atalaya de los Ojicos	ATA	47000	17.53	0.93	0.75	7.0	42.5
Hoya Rasa	HYR	40000	35.17	0.87	0.66	5.2	60.1
Hoya Chica	HYC	32000	10.79	0.51	0.12	5.5	71.6
La Campana	CAM	29000	4.9	0.63	0.11	2.9	61.6
Hoya del Monte	HMT	15800	9.36	0.51	0.19	5.6	83.1
Hoya Yerba	HYB	1060	5.03	0.23	0.34	3.5	82.7
Hoya Turnera	HTU	130	3.06	0.07	0.70	6.7	68.2

### *Brachionus plicatilis* genome

#### DNA extraction and DNA sequencing library construction

A monoclonal culture from one individual stem female hatched from a diapausing egg isolated from Hoya Rasa lake (HYR1 clone)^28^ was allowed to grow as described in Supplementary Method S2 to obtain enough biomass for DNA extraction. Genomic DNA was extracted with the DNeasy Blood and Tissue kit (Qiagen, Valencia, CA) following the manufacturer’s protocol. DNA quantification was performed using a dsDNA assay (Invitrogen) on a Qubit 2.0 fluorometer (Life Technologies) and quality was assessed by electrophoresis in a 1% agarose gel. For library construction, DNA was sheared through sonication down to ca. 550 bp by using a Diagenode Bioruptor. PCR-free DNA libraries were prepared with the sonicated DNA using the NEBNext Ultra DNA library Prep kit for Illumina combined with NEBNext Singleplex Adaptors. The library was quantified using a Qubit fluorometer and assessed by qPCR using the Illumina Library Quantification Kit (NEBNext Library Quant Kit) and a StepONE plus Real time PCR system (Applied Biosystems). The template DNA was denatured according to the protocol described in the Illumina system guide and loaded at 20 pM concentration. To improve sequencing quality, 1% PhiX control was spiked-in. The library was sequenced on a single Illumina MiSeq sequencer run with v3 chemistry (2 × 250 bp paired-end read lengths). Library preparation and sequencing was carried out at the EvoHull Genomics Lab at the University of Hull (Hull, United Kingdom).

#### Genome assembly and annotation

The sequenced reads were trimmed using Trimmomatic v.0.32^80^ to remove (1) Illumina adapters, (2) low quality leading or trailing regions (quality measured as Phred score; Phred score > 30) or unidentified nucleotides (i.e. Ns), (3) reads with average Phred score < 100, and (4) regions (5-base sliding window) with average Phred score < 20. Thereafter, reads were corrected using a Bloom filter-based error correction tool (BLESS v0.16)^81^. The paired reads were then merged using Usearch v. 8^82^. Pre-assembly assessment of k-mer distributions were obtained by KMC^83^ and profiling by GenomeScope^84^.

*De novo* genome assemblies were constructed with several assemblers using default parameters (Celera, SPAdes, DISCOVAR, and Platanus; Table 1) and discarding contigs shorter than 500 bp in the final assembly. Assembly quality was evaluated using (1) Blobtools^85,86^ to assess contamination, (2) CEGMA v. 2.5 (Core Eukaryotic Genes Mapping Approach)^87^, a tool that assesses genome completeness based on the presence of a set of core eukaryotic genes, and (3) Assemblathon scripts^88^ to obtain length-based statistics, such as N50. Platanus assembly was chosen as the reference assembly (see Results) and downstream analyses were performed on this assembly.

In order to perform the structural annotation of the assembly, GeneMark v. 4.32^89^ was initially used as an *ab initio* gene prediction software. After that, GeneMark predictions were provided to run MAKER2 v. 2.31.8^90^ an annotation pipeline that identifies repetitive elements, aligns expressed sequence tags (ESTs), and uses protein homology evidence to generate further gene predictions. MAKER2 was run twice. The first MAKER2 round was performed with permissive settings (Supplementary Data S1) using SNAP^91^ and RepeatMasker v.3.0^92^. In order to create evidence-based gene model predictions, MAKER2 was provided in this first run with: (1) the core eukaryotic gene models obtained from CEGMA, (2) ESTs from *B. plicatilis* NCBI database, (accession: 10-07-2016), (3) available transcriptomes from congeneric species (Supplementary Table S4), (4) proteins from *Adineta vaga* (a high-quality bdelloid rotifer genome)^54^, and (5) the Swiss-Prot database. The annotation output from MAKER2 first run was converted into a training set to run Augustus. Finally, the Augustus and MAKER2 (1^st^ pass) outputs were provided to perform a second MAKER2 pass with more stringent settings (Supplementary Data S2) in order to obtain the final set of gene predictions.

The functional annotation of the final set of predicted genes was performed by identifying protein domains with InterProScan v.5^93^ and by performing Blastp searches (e-value < 10^−5^)^94^ against a subset (metazoa only) of the NCBI’s non-redundant (nr) protein database. Finally, Blast2GO v.4.1.9^95^ was run with default settings to integrate results from BLAST and InterProScan and to retrieve Gene Ontology (GO) terms and annotation.

### Genotyping by sequencing of *B. plicatilis* clones

#### DNA extraction and DNA sequencing library construction

Each of the 270 clones of *B. plicatilis* was allowed to grow as described in Supplementary Method S2 to obtain enough biomass for DNA extraction. DNA extraction was performed with the JETFLEX Genomic DNA purification kit (GENOMED, Löhne, Germany) following manufacturer’s protocol. After quality assessment and quantification (see above), DNA samples were submitted to Cornell University Genomic Diversity Facility at the Institute of Biotechnology (IGD, Ithaca, NY, US), where libraries were constructed and sequenced. This was performed on an Illumina HiSeq. 2000/2500 (100 bp, single-end) according to Elshire *et al*.^45^, using the restriction enzyme ApeKI (GC[A-T]GC) for digestion. A library with unique barcodes for each clone, plus blank samples, was created.

#### SNP discovery and filtering

SNPs were called from the raw DNA sequences using the GBS pipeline as implemented in TASSEL-GBS v2 (TASSEL 5)^96^, in which all reads were trimmed to the same length (64 bp) and identical reads were collapsed into tags. These tags were then aligned against the reference genome using the Burrows-Wheeler alignment tool (BWA)^97^ and SNPs were called from aligned tags. Pipeline default parameters were used except for two cases, in which more conservative values than default were used: (1) at the Minimum length of aligned base pair to store the Sequence Alignment/Map (SAM) entry (SAMToGBSdbPlugin plugin, option aLen being 30 instead of default 0); and (2) at the Minimum locus coverage (i.e. proportion of Taxa with a genotype; DiscoverySNPCallerPluginV2 plugin, option mnLCov being 0.8 instead of default 0.1).

The set of SNPs were quality filtered using custom developed scripts (Supplementary Data S3) and VCFtools^98^. In order to obtain confidently assigned genotypes, those genotypes at each sample supported by less than six reads were excluded (considered as not genotyped). A set of filters were applied to retain those SNPs highly informative, of high quality and not representing over-merged repetitive regions. Firstly, SNPs required at least 50% of the clones genotyped in each population. Second, minor allele frequency (MAF) had to be higher than 1%. Third, only two alleles were present. Fourth, the average read depth among clones had to be lower than 150 reads^48^. Fifth, less than 60% of clones had to be heterozygotes in each SNP.

#### Population genomic analyses

To estimate genome-wide genetic variation and differentiation, principal component analysis (PCA), a model-free multivariate ordination method, was performed as implemented in the “adegenet” package^99^ in R v.3.3.3^100^. Using the package “hierfstat”^101^, genetic divergence between populations was estimated by the fixation index (*F*_*ST*_) for each pairwise comparison, and within-population genetic diversity was measured as the average among loci expected heterozygosity (*H*_*e*_), observed heterozygosity (*H*_*o*_) and inbreeding coefficient (*F*_*IS*_). Mantel tests were used to test for isolation-by-distance patterns in IBDWS v.3.23^102^ using the geographic distance between lakes calculated using Google Earth (2008) and pairwise *F*_*ST*_ as genetic distance. The relationship between heterozygosity (*H*_*e*_) and log-transformed lake area (Table 5) was tested using correlation analysis. Tajima D-values and Hardy-Weinberg tests were obtained using Plink v. 1.90^103^. File format conversions were performed using PGDSpider v. 2.1.0.3^104^.

#### Putative SNPs under selection and associated genes

To identify SNPs likely to be differentiated as a result of selection, three different approaches were used. Firstly, BayeScan 2.1^105^ was used to estimate the posterior probability that a given SNP is affected by selection based on the allele frequencies in each population. It identifies those SNPs with significantly higher —i.e. diversifying selection– or lower —i.e. balancing/purifying selection– differentiation among populations than expected under a model of neutral evolution^18^. Briefly, prior odds of 10 were used to identify the top candidate SNPs under selection with a false discovery rate of 0.05. Secondly, we used Bayenv2^106^ to identify loci correlated with particular environmental and phenotypic variables. Bayenv uses a Bayesian approach that takes into account population structure -from covariance matrices- when testing for correlations between environmental and population allelic frequencies^106^. To do so, the full set of SNPs were used for the construction of 10 covariance matrices using different priors (following Hahn *et al*.)^107^. The association with genetic variation was tested for three environmental variables known for the lakes under study (environmental predictability, hydroperiod length and salinity)^26^, and two phenotypic variables previously estimated for the genotyped populations (life-history traits: propensity for sex and hatching fraction, Table 5)^28^. For the latter, we used the average of the clones genotyped here for the corresponding population. Each environmental or phenotypic variables was standardized by subtracting the mean and dividing by the standard deviation of the variable. Twenty independent runs with different random seeds were run, obtaining for each SNP the Bayesian Factors (a parameter usually used in Bayesian statistics for model choice, hereafter BF). Following Hancock *et al*.^108^ we used the BF of all of the SNPs to calculate a rank statistic (each SNP scaled between 0 and 1; corresponding to the SNP with the lowest and highest BF per run, respectively). The rank statistic was averaged between runs to obtain the mean rank of each SNP. SNPs were considered as potentially under selection when their mean rank was above 0.99. Thirdly, a genome-wide association study (GWAS) was performed for both life-history traits in order to explore genotype-phenotype association by using GenABEL R package^109^ with EIGENSTRAT methodology^110^. In this approach the inter-clonal variability is analysed by considering individual phenotypic values and genotypes, which was hidden behind the averages used in the Bayenv approach. This methodology uses PCA to correct for population stratification. For each SNP and phenotypic variable association analyzed, it provides the Bonferroni adjusted *p*-value and the *R*^2^. SNPs with p-value lower than 0.05 are considered to be putatively associated to the phenotype.

In order to identify the genes associated to putative SNPs under selection —as identified by BayeScan, Bayenv or GenABEL–, BEDtools v.2.19.1^111^ was used to find genes in a flanking region of 0, 2.5 or 5 Kb upstream and downstream from the focal SNPs. As expected from the compact genome of *B. plicatilis* (Table 2), a large number of putative genes were found closely linked to SNPs under selection (Supplementary Table S2). A GO enrichment analysis (Fisher’s exact test, two- tailed, false discovery rate < 0.05) was conducted to test if certain gene ontologies were over- or under- represented in the lists of genes putatively under selection with respect to the genome using Blast2GO v. 4.1.9^95^.

The sequences used in this study were deposited in GenBank (BIOproject ID: PRJNA422189). GBS raw sequences were deposited in the ENA (accession number SRP151997). The Whole Genome Shotgun project has been deposited at DDBJ/ENA/GenBank under the accession REGN00000000. The version described in this paper is version REGN01000000.

## Electronic supplementary material

Supplementary material file information

Dataset 1

Dataset 2

Dataset 3
