# Supplementary material for: Genomic signatures of local adaptation to the degree of environmental predictability in rotifers
Source: Sci Rep. 2018 Oct 30;8:16051. doi: 10.1038/s41598-018-34188-y (PMC6207753; doi:10.1038/s41598-018-34188-y)

# Genomic signatures of local adaptation to the degree of environmental predictability in rotifers

Lluis Franch-Gras, Christoph Hahn, Eduardo M. García-Roger, María José Carmona, Manuel Serra and Africa Gómez

Supplementary Method S1: Definition of standard conditions.

Clones were maintained in 15 mL stock cultures at 12 g $L^{-1}$ salinity, 20ºC and *Tetraselmis suecica* as food.

Supplementary Method S2: Protocol to grow the clone cultures in order to obtain enough biomass for DNA extraction.

The clone was allowed to grow under the standard conditions specified above for 15 days, starting from low density stock culture. During the first 5 days, rotifers were kept in a flask containing 200 mL of culture medium and, after that, they were transferred to eight bottles containing 1.5 L of culture medium. On day 10, when >10,000 individuals were reached in each bottle, rotifers were filtered out through a 30-µm Nytal mesh sieve and released on saline water 12 g $L^{-1}$ for 24 hours in order to purge the rotifer's digestive tracts and thus minimize contamination with microalgae DNA. Finally, the animals were concentrated by filtering again with a new 30-µm Nytal mesh sieve to obtain the biomass needed for genomic DNA extraction.

Supplementary Data S1: File with the options provided to Maker2 with permissive settings.

Supplementary Table S1: Number of SNPs shared between each of the variables studied in Bayenv and the SNPs found to be candidates for diversifying selection (Bayescan). No SNPs candidate for being under balancing selection was also associated to any other variable.

| | ***Diversifying selection*** | ***Propensity for sex*** | ***Hatching fraction*** | ***Environmental predictability*** | ***Hydroperiod length*** | ***Salinity*** |
|---|---|---|---|---|---|---|
| Diversifying selection | 12 | | | | | |
| Propensity for sex | 1 | 37 | | | | |
| Hatching fraction | 2 | 6 | 38 | | | |
| Environmental predictability | 0 | 10 | 4 | 34 | | |
| Hydroperiod length | 0 | 4 | 7 | 6 | 39 | |
| Salinity | 1 | 2 | 10 | 2 | 5 | 39 |

Supplementary Table S2: Number of genes detected to be physically associated to SNPs under selection. Results for (1) three window sizes around each SNP and (2) three approaches to detect selection are reported.

| **Approach** | **Type of selection/ Associated variable** | **Number of SNP** | **Window size** | | |
|---|---|---|---|---|---|
| | | | **0 Kb** | **2.5 Kb** | **5 Kb** |
| **BayeScan** | **Balancing** | 81 | 39 | 144 | 199 |
| | **Diversifying** | 12 | 12 | 37 | 49 |
| **Bayenv** | **Propensity for sex** | 37 | 34 | 95 | 139 |
| | **Hatching fraction** | 38 | 35 | 94 | 127 |
| | **Predictability** | 34 | 27 | 78 | 119 |
| | **Hydroperiod** | 39 | 28 | 80 | 119 |
| | **Salinity** | 39 | 27 | 72 | 114 |
| **GenABEL** | **Propensity for sex** | 1 | 1 | 1 | 1 |
| **Overall** | | 261 | 164 | 483 | 696 |

Supplementary Table S3: Significantly overrepresented gene ontologies (GO) in the genes associated to the variables studied (Fisher’s exact test, two- tailed, false discovery rate (FDR) < 0.05).

| ***Variable*** **GO ID** | **GO Category** | **GO Name** | **FDR** | ***p*-value** |
|---|---|---|---|---|
| *Hatching fraction* | | | | |
| GO:0004871 | MOLECULAR FUNCTION | signal transducer activity | 2.80E-02 | 3.90E-06 |
| GO:0005488 | MOLECULAR FUNCTION | binding | 1.08E-07 | 1.50E-11 |
| GO:0097159 | MOLECULAR FUNCTION | organic cyclic compound binding | 3.60E-04 | 1.50E-07 |
| GO:1901363 | MOLECULAR FUNCTION | heterocyclic compound binding | 3.60E-04 | 1.49E-07 |
| GO:0005524 | MOLECULAR FUNCTION | ATP binding | 6.29E-04 | 5.57E-07 |
| GO:0030554 | MOLECULAR FUNCTION | adenyl nucleotide binding | 6.29E-04 | 6.13E-07 |
| GO:0043168 | MOLECULAR FUNCTION | anion binding | 6.29E-04 | 4.64E-07 |
| GO:0032559 | MOLECULAR FUNCTION | adenyl ribonucleotide binding | 6.29E-04 | 6.09E-07 |
| GO:0001882 | MOLECULAR FUNCTION | nucleoside binding | 1.35E-03 | 2.61E-06 |
| GO:0001883 | MOLECULAR FUNCTION | purine nucleoside binding | 1.35E-03 | 2.49E-06 |
| GO:0017076 | MOLECULAR FUNCTION | purine nucleotide binding | 1.35E-03 | 2.63E-06 |
| Environmental predictability | | | | |
| GO:0035639 | MOLECULAR FUNCTION | purine ribonucleoside triphosphate binding | 1.35E-03 | 2.37E-06 |
| GO:0032555 | MOLECULAR FUNCTION | purine ribonucleotide binding | 1.35E-03 | 2.55E-06 |
| GO:0032550 | MOLECULAR FUNCTION | purine ribonucleoside binding | 1.35E-03 | 2.48E-06 |
| GO:0032549 | MOLECULAR FUNCTION | ribonucleoside binding | 1.35E-03 | 2.58E-06 |
| GO:0032553 | MOLECULAR FUNCTION | ribonucleotide binding | 1.35E-03 | 2.83E-06 |
| GO:0097367 | MOLECULAR FUNCTION | carbohydrate derivative binding | 2.03E-03 | 4.68E-06 |
| GO:0043167 | MOLECULAR FUNCTION | ion binding | 2.03E-03 | 4.82E-06 |
| GO:1901265 | MOLECULAR FUNCTION | nucleoside phosphate binding | 5.17E-03 | 1.37E-05 |
| GO:0000166 | MOLECULAR FUNCTION | nucleotide binding | 5.17E-03 | 1.37E-05 |
| GO:0036094 | MOLECULAR FUNCTION | small molecule binding | 5.77E-03 | 1.61E-05 |
| GO:0003676 | MOLECULAR FUNCTION | nucleic acid binding | 7.15E-03 | 2.09E-05 |
| Propensity for sex | | | | |
| GO:0005488 | MOLECULAR FUNCTION | binding | 0.01 | 2.36E-06 |
| O:0050794 | BIOLOGICAL PROCESS | regulation of cellular process | 0.01 | 3.08E-06 |
| *Salinity* | | | | |
| GO:0050789 | BIOLOGICAL PROCESS | regulation of biological process | 0.01 | 3.61E-06 |
| GO:0065007 | BIOLOGICAL PROCESS | biological regulation | 0.01 | 6.83E-06 |

Supplementary Table S4: Transcriptomes used in the Brachionus plicatilis genome assembly from congeneric species.

| **Species** | **Accession number** | **Reference** |
|---|---|---|
| *B. manjavacas* | GARS01000001-GARS01014244 | Welch et al. (2014) |
| *B. calyciflorus* | GACL00000000.1 and GACQ00000000.1 | Hanson et al. (2013) |
| *B. koreanus* | GBXV00000000 | Lee et al. (2015) |

Supplementary Data S2: File with the options provided to Maker2 with more stringent settings.

Supplementary Data S3: Ipython notebook used to quality filter the SNPs.

Supplementary Figure S1: K-mer plot and statistics for filtered and error-corrected raw data obtained from genome sequencing (k-mer length= 21).

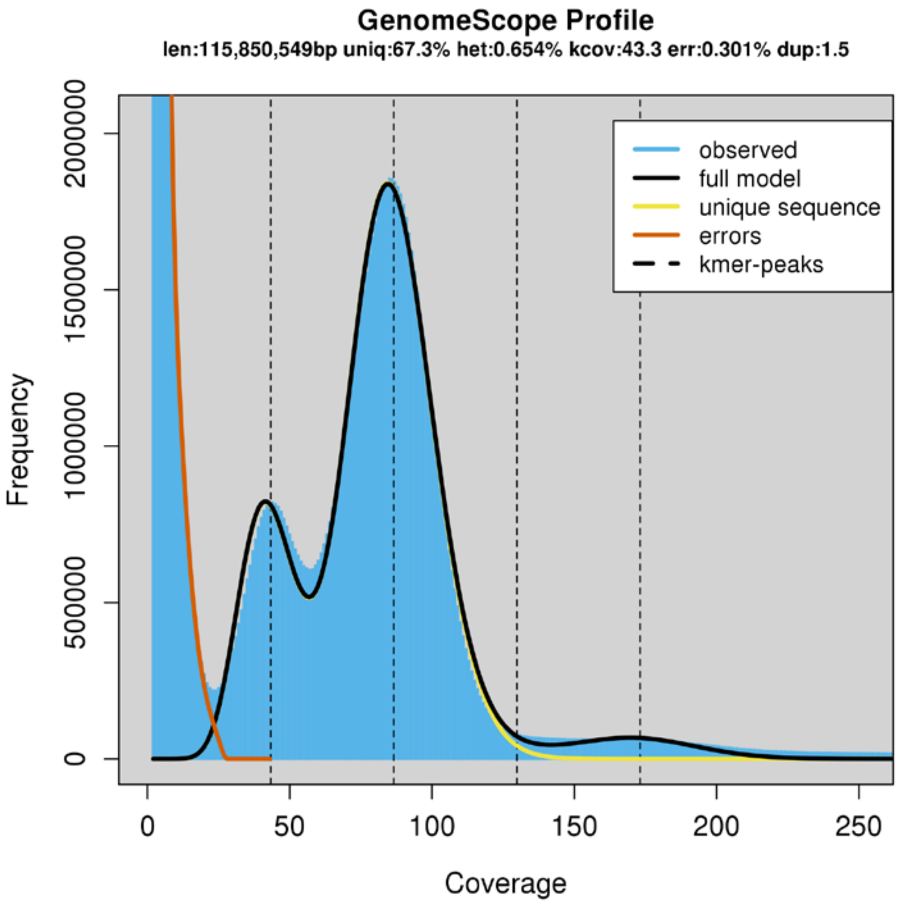


| Property | min | max |
|---|---|---|
| Heterozygosity | 0.653566% | 0.658388% |
| Genome Haploid Length | 115,775,183 bp | 115,850,549 bp |
| Genome Repeat Length | 37,904,922 bp | 37,929,597 bp |
| Genome Unique Length | 77,870,261 bp | 77,920,952 bp |
| Model Fit | 90.069% | 93.868% |
| Read Error Rate | 0.301464% | 0.301464% |

Supplementary Figure S2: Blobplot of the draft genome of *Brachionus plicatilis* based on G+C content and contig coverage.

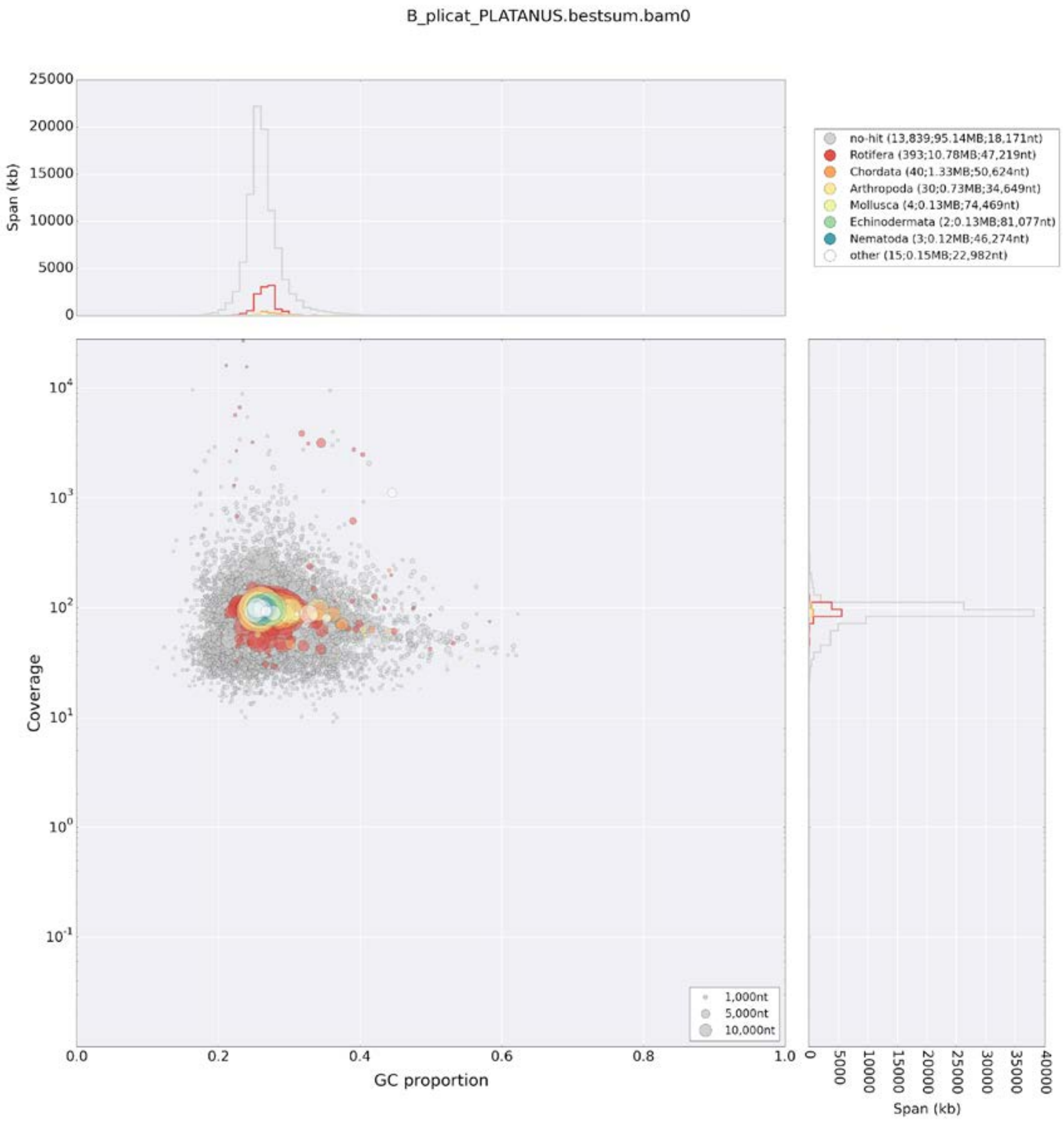

Supplementary Figure S3: Boxplots showing the distribution of the coverage of the SNPs when: a) all SNPs were considered; b) Only those SNPs putatively under balancing/purifying selection or c) Only those that are putatively under diversifying selection.

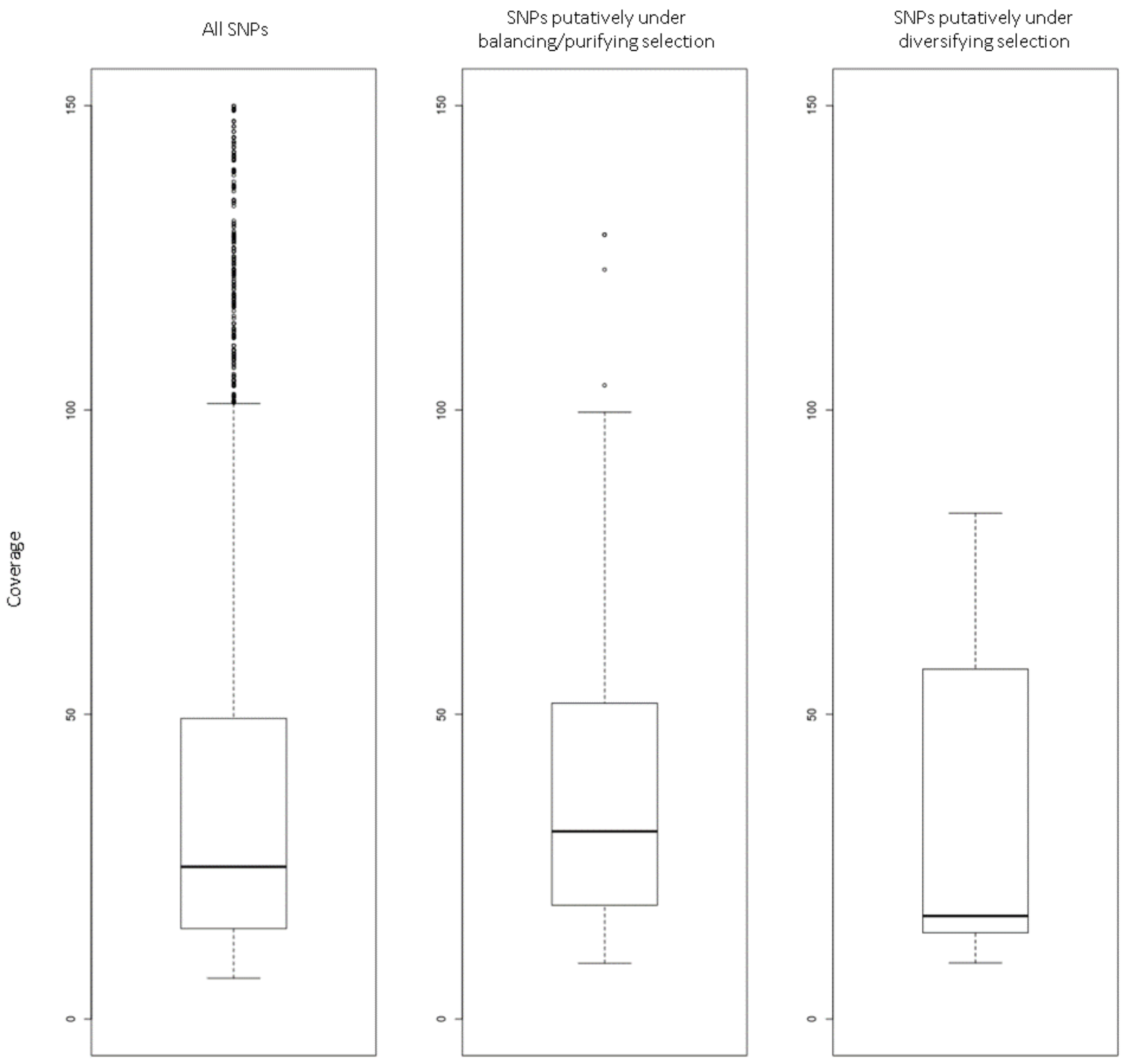

Supplementary Figure S4: Within population linkage disequilibrium analysis between each pair of SNPs. X axis represents the genetic distance and Y axis represents the $R^2$. This analysis was performed using Plink (for references, see the main body).

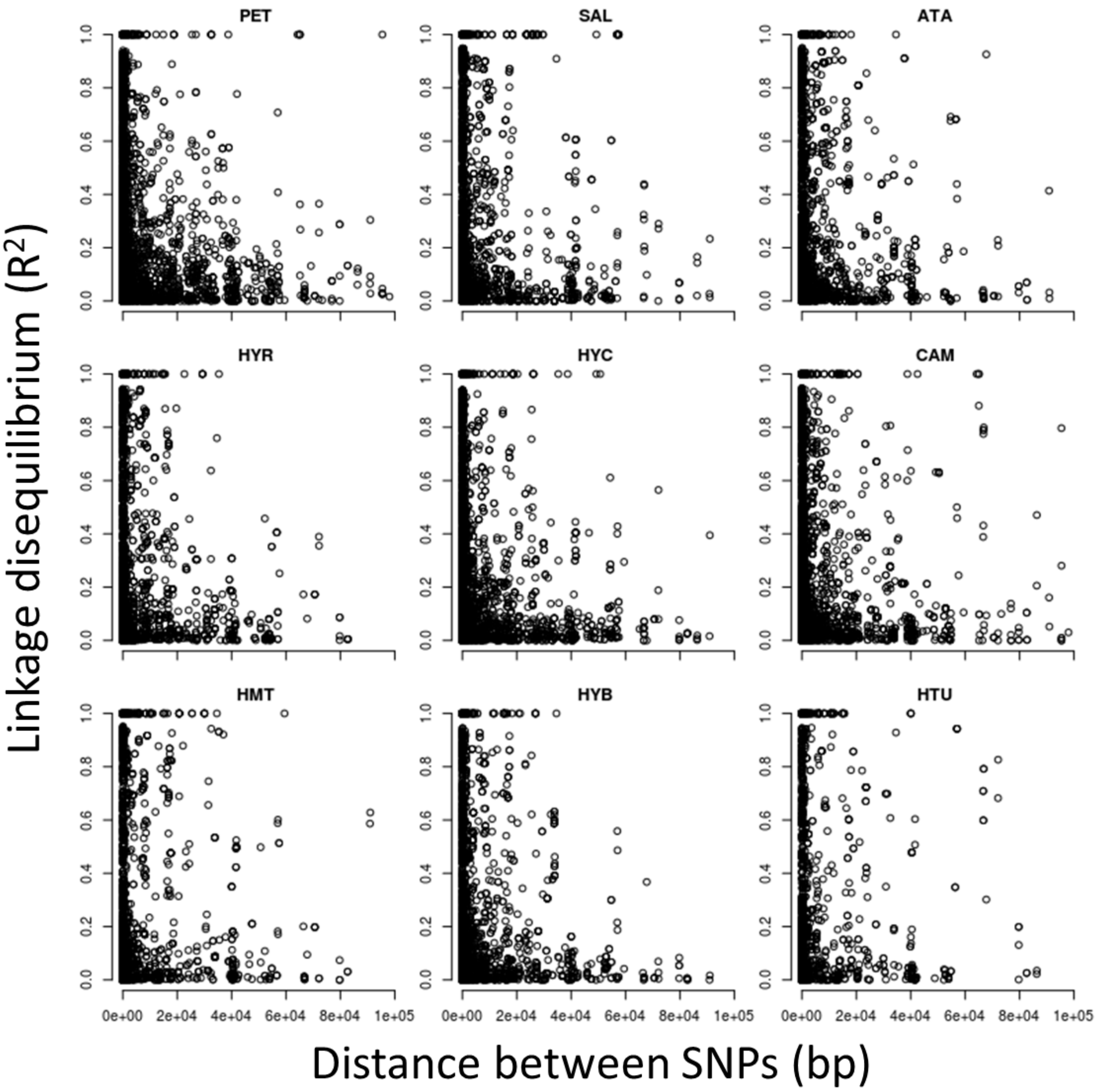

Supplement: Supplementary file 1 — Supplementary material file information [file 41598_2018_34188_MOESM1_ESM.pdf]
